# Patients with hip resurfacing arthroplasty are not physically more active than those with a stemmed total hip

**DOI:** 10.1080/17453674.2020.1771652

**Published:** 2020-06-04

**Authors:** Jetse Jelsma, Martijn G M Schotanus, Ivo T A F Buil, Sander M J van Kuijk, Ide C Heyligers, Bernd Grimm

**Affiliations:** a Department of Orthopedic Surgery and Traumatology, Zuyderland Medical Centre, Sittard-Geleen, The Netherlands;; b Department of Innovation and Funding, Zuyderland Medical Centre, Sittard-Geleen, The Netherlands;; c Department of Clinical Epidemiology and Medical Technology Assessment (KEMTA), Maastricht, The Netherlands;; d School of Health Professions Education, Maastricht University, Maastricht, The Netherlands;; e Luxembourg Institute of Health, Human Motion, Orthopedics, Sports Medicine, Digital Methods (HOSD), Luxembourg, Luxembourg

## Abstract

Background and purpose — Hip resurfacing arthroplasty (HRA) was designed for the highly active patient because of the various theoretical advantages compared with stemmed total hip arthroplasty (THA), but has shown high failure rates. Physical activity (PA) after arthroplasty is frequently determined with the use of questionnaires, which are known for their subjective nature, recall bias, and ceiling effect. These disadvantages are not applicable to physical activity monitoring (AM) using sensors. We compared objectively measured PA at long-term follow-up in a matched cohort of HRA and stemmed THA subjects.

Patients and methods — We compared 2 groups of 16 patients (12 males) in each group, one having received unilateral HRA (median age 56 years at surgery) and a matched group having received unilateral stemmed THA with a small diameter femoral head (28 mm) on conventional polyethylene (median age 60 years at surgery) with osteoarthritis as indication for surgery, 10 years after surgery. Groups were matched by sex, age at surgery, and BMI. The daily habitual PA was measured over 4 consecutive days in daily living using a 3-axis accelerometer, gyroscope, and magnetometer. Both quantitative parameters (time standing, sitting, walking, number of steps, and sit–stand transfers) and qualitative parameters (walking cadence) were determined.

Results — The AM was worn for a median 13 (11–16) hours per day. The median daily step count was 5,546 (2,274–9,966) for the HRA group and 4,583 (1,567–11,749) for the stemmed THA-group with 39 (21–74) versus 37 (24–62) daily sit–stand transfers respectively. The other PA parameters were also similar in both groups.

Interpretation — We found similar median PA levels and also identical ranges. While short-term effects may exist, ageing and related behavioral adaptations or other effects seem to render the theoretical activity benefits from HRA irrelevant at longer follow-up.

Metal-on-metal (MoM) hip resurfacing arthroplasty (HRA) was designed for the highly active patient because of various theoretical advantages compared to conventional stemmed metal-on-polyethylene (MoP) THA: low volumetric wear, large physiological diameter femoral heads offering stability, near-natural joint kinematics, and increased range of motion compared with small-diameter THA and preservation of the femoral bone (Corten et al. 2011). HRA was commonly advertised as “the sporting hip” and publicity was created with subjects participating in triathlons after HRA (Girard et al. 2017). Gait analysis studies showed that HRA subjects returned to a more normative gait pattern with a higher walking speed when compared with THA (Nantel et al. 2009, Gerhardt et al. 2019). HRA showed initially promising results; however, since 2004 concerns have been raised because of high failure rates. In the Netherlands the use of HRA has been forbidden by the Dutch Orthopaedic Society (NOV) and Government since January 2012 (Verhaar 2012).

It can be expected that patients who received an HRA would be more physically active after surgery when compared with patients who received a stemmed THA with a small-diameter MoP or ceramic-on-polyethylene (CoP) bearing for multiple reasons: the theoretically better implant design features of HRA listed above claiming to support a more active lifestyle, the selection of patients for this particular implant (young and active), and the related patient expectations (high preoperative demands on postoperative activity). A few studies have investigated return to sports after HRA and showed that patients were able to return to high activity levels and sporting activities postoperatively, at least at short-time follow-up (Narvani et al. 2006, Naal et al. 2007). Some studies comparing an HRA with a stemmed MoP or CoP THA showed higher postoperative activity levels after HRA at 3–4 years’ follow-up. However, activity levels were determined using a (weighted) self-reported activity questionnaire (Zywiel et al. 2009, Mont et al. 2009). The highly subjective nature, strong recall bias, and possible ceiling effect are known disadvantages of such questionnaires, especially for quantifying activity levels, which is in contrast to activity monitoring (AM) using sensors (Terwee et al. 2011). Wearable AMs measure a patient’s habitual physical activity (PA) objectively and continuously in the free-living environment and different physical activities can be differentiated (Lipperts et al. 2017, Jelsma et al. 2019).

We objectively measured PA at long-term follow-up in an age- and sex-matched cohort of HRA and stemmed THA subjects. The hypothesis was that subjects with a unilateral HRA are physically more active in habitual daily life, measured by AM.

## Patients and methods

We conducted a cohort study at the Zuyderland Medical Centre, Sittard-Geleen, The Netherlands between August and November 2017 (recruitment of HRA group) and from February to June 2018 (recruitment of stemmed THA group). We compared 2 groups, one having received unilateral HRA and a matched group having received unilateral stemmed THA with a small diameter metal or ceramic femoral head (28 mm) on conventional polyethylene with osteoarthritis as indication for surgery. The HRA group with a median follow-up of 10 (9–11) years was initially recruited for another study, the methods of which are described in detail elsewhere (Jelsma et al. 2020). The stemmed THA group was matched by sex, age at surgery, follow-up since surgery, and BMI. Patients with an uncemented, unilateral stemmed THA with a MoP or CoP bearing were included. The follow-up was set at 8–12 years to optimize the chances of a matched cohort. Finally, 16 patients consented to the study and were included as a matched cohort (Figure). There were no statistically significant differences between group characteristics at baseline (Table 1). The use of the AM has been described in detail elsewhere (Lipperts et al. 2017). The daily habitual PA was measured during waking hours for 4 consecutive days in daily living. The AM used to collect the raw signal was a 3-axis accelerometer, gyroscope, and magnetometer (HAM-IMU + alt, Gulf Coast Data Concepts LLC, Waveland, MI, USA). The data received with this AM were analyzed with MATLAB (MATLAB R2017a, The Mathworks Inc., Natick, MA, USA) with the use of previously validated algorithms with excellent accuracy (> 97%) in determining PA levels in a semi-free setting (Lipperts et al. 2017). With the AM, various quantitative parameters of PA can be obtained and in this study we assessed the following metrics: the time in hours standing, sitting, walking, and cycling and the amount of steps and sit–stand transfers. Walking cadence, defined as the number of steps per minute and a proxy of walking speed, was calculated as a qualitative parameter.

**Figure:**
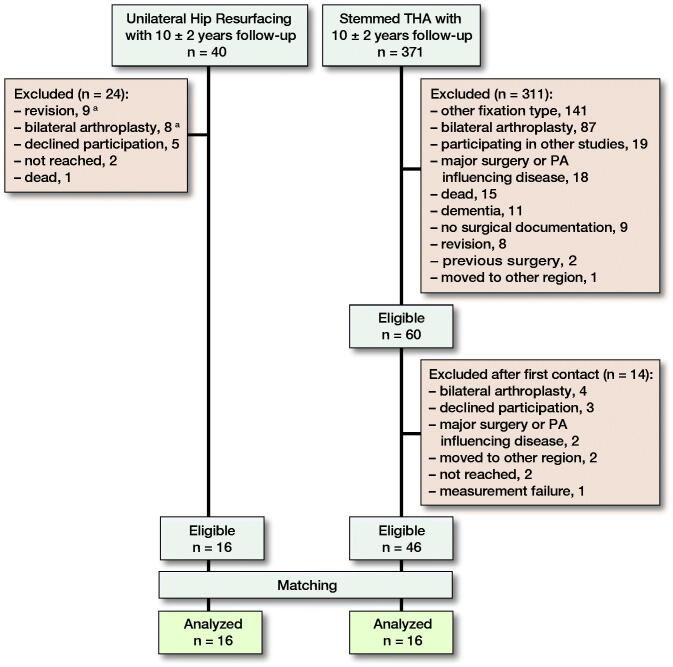
Number of patients enrolled and analyzed in this study. **^a^**One patient underwent revision surgery, but died 6 months later due to a non-surgical reason. 1 patient underwent revision surgery and a primary arthroplasty contralaterally a few years after.

**Table 1.** Baseline characteristics. Values are median (range) unless otherwise specified

Factor	HRA group	Stemmed THA group	p-value
Male sex, n	12	12	1.0
Age at surgery	56 (43–67)	60 (53–68)	0.1
Follow-up (years)	10 (9–11)	10 (8–12)	0.4
BMI	26 (22–37)	29 (20–40)	0.1
Approach, n			1.0
Posterolateral	7	7	
Straight lateral	9	9	
Bearing, n			
MoM	16	–	–
MoP	–	4	–
CoP	–	12	–

MoM = metal-on-metal, MoP = metal-on-polyethylene,

CoP = ceramic-on-polyethylene.

We also assessed outcome by 3 commonly used patient-reported outcome measures (PROMs): the 12-item Forgotten Joint Score (FJS-12) and the Hip disability and Osteoarthritis Outcome Score Physical Function Short Form (HOOS-PS), both with “100” as the best possible score, and the Short Questionnaire to Assess Health-enhancing physical activity (SQUASH) to determine a total activity score (Davis et al. 2008, Behrend et al. 2012, Wendel-Vos et al. 2003).

### Statistics

Group comparison (e.g., patient characteristics) and parameters of PA between the groups were performed using the Mann–Whitney U test, because of the small groups, Pearson’s chi-square test and, in the case of expected cell-counts, Fisher’s exact test was used to test for differences between groups present at baseline. For all analyses, a p-value was considered to be statistically significant at p ≤ 0.05. Results are presented as median (range). IBM SPSS Statistics 22 (IBM Corp, Armonk, NY, USA) was used for statistical analysis.

### Ethics, funding, and potential conflicts of interest

This study was performed in compliance with the 1975 Declaration of Helsinki, as revised in 2013, and was studied and approved by the IRB (METC Zuyd, Heerlen, The Netherlands, IRB nr: 10N72 + amendment) and conducted in accordance with the guidelines for Good Clinical Practice (GCP). All patients signed informed consent. The authors declare they do not have any kind of conflict of interest.

## Results

Both groups had similar baseline characteristics (Table 1) and showed similar PA monitor parameters (Table 2) and PROMs (Table 3).

**Table 2.** Parameters of physical activity monitoring. Values are median (range)

Factor	HRA group	Stemmed THA group	p-value
AM wearing time (h)	12 (11–16)	14 (11–16)	0.1
Time sitting (h)	7.6 (4.6–12)	9.6 (3.8–13)	0.1
Time standing (h)	3.0 (1.8–5.7)	3.0 (1.6–6.2)	0.9
Time walked (h)	1.3 (0.5–1.9)	1.1 (0.4–1.8)	0.6
Time cycled (h)	0.05 (0.0–0.48)	0.01 (0.0–1.2)	0.5
Steps taken (nЧ10^3^)	5.5 (2.3–10)	4.6 (1.6–11)	0.6
Sit–stand transfers (n)	39 (21–74)	37 (24–65)	0.7
Cadence (steps/min)	102 (81–112)	98 (80–110)	0.3

**Table 3.** Patient reported outcome measures. Values are median (range)

Factor	HRA group	Stemmed THA group	p-value
HOOS-PS	87 (49–100)	67 (49–100)	0.3
FJS-12	63 (2–100)	56 (4–100)	0.5
SQUASH	6,150 (1,110–18,480)	4,560 (1,050–9,300)	0.2

## Discussion

This observational matched-cohort study showed that patients with a unilateral HRA are not physically more active when compared with subjects with a unilateral stemmed MoP or CoP THA at 10 years’ follow-up. This is counterintuitive to expectations, as HRA patients received the theoretical advantages of the implant design (large, near physiological head diameter) and surgical procedure (anatomical preservation), and represent a selection bias towards subjects presenting, being perceived by the surgeon, or themselves expecting to be more physically active and demanding than patients in the stemmed conventional MoP/CoP small-diameter head THA group. In addition, in this matched-cohort study, the median age and BMI was higher, but not significant, in the stemmed THA group, both factors established to be related with a less active lifestyle. Patients with both types of implants did not only have comparable mean PA levels but also showed identical ranges. Thus it seems that both implant types enable the same level of PA and that activity levels depend on individual lifestyle rather than on implant type, at least at 10 years’ follow-up.

PA is considered a major risk factor for a number of adverse health outcomes. Reaching a daily step count > 8,000 has been associated with a lower risk of all-cause mortality (Saint-Maurice et al. 2020). In our study 13 subjects (5 HRA, 8 stemmed THA) made < 5,000 steps/day, 5 subjects ≥ 8,000 steps/day (2 HRA, 3 stemmed THA), and only 1 subject > 10,000 steps/day (HRA). This suggest that almost half of the patients in this study would be considered to be living sedentary lifestyles with the associated risks of developing non-communicable diseases (Tudor-Locke et al. 2011). Sedentary time as a parameter is related to but largely independent of PA levels and was numerically higher in THA than HRA (p = 0.05), but this absolute time difference corresponds almost completely to the difference in total wear time between both groups, indicating a difference in instructions or compliance with it for wear time (waking hours) more than in activity behavior.

The groups showed no statistically significant differences in the HOOS-PS and FJS-12, suggesting that the groups are comparable according to the assessed domains such as pain, patient satisfaction, or perceived function. A 20-points mean difference was seen in the HOOS-PS in favor of HRA. This is in accordance with a recent publication of Oxblom et al. (2019). They studied 726 subjects 7 years after primary HRA or conventional THA showing a significant difference in HOOS subscales of function of daily living and function in sport and recreation, although HOOS subscales of symptoms, pain, and quality of life, EQ-5D index, and visual analog scores for pain and satisfaction did not differ.

It has been shown that patients 1 year after receiving stemmed THA show only a few changes in objectively measured free-living PA compared with preoperative levels (Jeldi et al. 2017, Thewlis et al. 2019). While the reason not to use a pain-free hip and improved functional capacity towards higher PA levels is multifactorial, one possible explanation is that, as PA levels are known to be related to wear of MoP bearings, long-term participation in high-impact activities is usually not recommended (Schmalzried et al. 2000). However, there is little prospective evidence reporting a poor clinical outcome with higher levels of activity (Meira and Zeni 2014). In Danish and American guidelines and a Dutch survey most low-impact activities only were allowed, though not necessarily promoted post-THA (Meester et al. 2018). This is in contrast to the advice given by orthopedic surgeons to HRA patients and the publicity of HRA manufacturers calling it a “sporting hip.” Multiple studies have shown a high return to sport, including high-intensity activities such as long-distance triathlon, after HRA (Fouilleron et al. 2012, Girard et al. 2017). For subjects with a conventional THA this has not been advised but may be possible.

The studies in current literature comparing PA or sports participation in HRA and stemmed THA have all been performed using (weighted) PA questionnaires (Mont et al. 2009, Zywiel et al. 2009). Our study is one of the first of its kind to evaluate habitual PA in the free-living environment using a wearable AM. Questionnaires are limited by the highly subjective nature and ceiling effect, which is in contrast to the objective results obtained by AM (Terwee et al. 2011). Zywiel et al. (2009) performed a study comparing PA, measured by a weighted questionnaire (comparable with the SQUASH), in HRA and a matched cohort of patients with a stemmed THA. At final follow-up (3–4 years) the HRA group had a higher mean weighted activity score than the stemmed THA group (p < 0.01), while activities preoperatively were similar. It was not stated whether there were differences regarding the instructions for postoperatively approved (high-intensity) PA. Other comparison studies by Pollard et al. (2006), Vail et al. (2006) and Mont et al. (2009) used UCLA activity scores and weighted activity scores and found a higher degree of PA in HRA, although these studies have numerous limitations, mainly related to the uncontrolled bias of HRA towards very high preoperative PA levels.

Our study has limitations. The number of subjects was rather low. The main cause for this was the initially strict inclusion criteria for the initial HRA study (Jelsma et al. 2020). Another limitation was that no objective information, e.g., PA monitoring data, were available for the preoperative setting of the subjects. This might have influenced our results, because the physically more active patients could have been designated for hip resurfacing at the time of surgery, and therefore selection bias may have occurred. However, such a possible selection bias would further support the findings of this study.

### Conclusion

This is the first study comparing postoperative PA levels between HRA and stemmed THA using wearable sensors for objective PA measures. HRA theoretically supports high PA levels, by design and surgery, which should result in a difference at 10 years, although this study found no differences in PA and ranges are also comparable. Even well-reasoned theoretical advantages concerning functional advantages of any implant design require clinical validation and should not be assumed as an indication (especially at the risk of a disadvantage). While short-term effects may exist, ageing and related behavioral adaptations or other effects seem to render the theoretical activity benefits from HRA irrelevant at longer follow-up. PA levels at long follow-up seem to depend less on implant type but rather on other factors, warranting further research to ensure the related health benefits in THA patients.
